# Biomarkers to Detect Early-Stage Colorectal Cancer

**DOI:** 10.3390/biomedicines10020255

**Published:** 2022-01-25

**Authors:** Jacqueline I. Keenan, Frank A. Frizelle

**Affiliations:** Department of Surgery, University of Otago Christchurch, Christchurch 8140, New Zealand; frank.frizelle@otago.ac.nz

**Keywords:** colorectal cancer, biomarker, diagnosis, early stage, noninvasive testing

## Abstract

Colorectal cancer is a leading cause of mortality worldwide. The high incidence and the acceleration of incidence in younger people reinforces the need for better techniques of early detection. The use of noninvasive biomarkers has potential to more accurately inform how patients are prioritised for clinical investigation, which, in turn, may ultimately translate into improved survival for those subsequently found to have curable-stage CRC. This review surveys a wide range of CRC biomarkers that may (alone or in combination) identify symptomatic patients presenting in primary care who should be progressed for clinical investigation.

## 1. Introduction

Colorectal cancer (CRC) is a leading cause of mortality worldwide. While western countries continue to have the highest rates of CRC, acceleration of CRC incidence is increasingly reported in in newly industrialised countries as these societies have become more westernised [1]. Most of these cancers are considered sporadic and current thinking is that their development is driven by an environmental and/or lifestyle factor(s) that promotes chronic inflammation and/or DNA damage with associated changes in the microcellular environment, resulting in pre-cancerous and finally cancerous changes [2]. This is reinforced by the observation that people who migrate from low- to high-risk areas of the world rapidly assume the CRC risk of the host country [3], A growing awareness of the rapidly increasing burden on left sided cancers in those under 50, that looks likely to result in 1 in 4 rectal cancers being found in patients under 50 in ten years’ time [4] gains significance as diets in developing countries become increasingly westernised [5].

CRC is usually surgically curable in the early stages (I and II) of the disease, with a 5-year relative survival of about 90% [6]. The key to a good prognosis is early diagnosis. This remains an issue however, not least because patients with early-stage disease have no symptoms or present with nonspecific symptoms in primary care may require multiple visits before referral for investigation. This is of particular concern in young patients. While colonoscopy remains the gold standard procedure used to formulate the diagnosis of CRC based on endoscopic and histopathological assessment, the availability of endoscopy time in most public health systems is limited, resulting in triaging of referrals for colonoscopy. The nonspecific nature of many gastrointestinal symptoms means many patients have a normal colonoscopy [7]. Increasingly, the use of noninvasive biomarkers is being investigated for their potential to more accurately inform how patients are prioritised for clinical investigation, which, in turn, may ultimately translate into improved survival for those subsequently found to have curable-stage CRC.

These biomarkers include proteins and metabolites released by various cells during an active disease state, or more complex biomarkers involving multilevel “omics” to detect cell-associated genetic markers and/or dysbiosis of gut microbiota. They are assessed in a range of biological samples including blood, faeces, urine, breath and, more recently, colonic mucus [8]. Ideally, biomarker testing should be easily performed and relatively inexpensive [9]. However, if biomarkers are to accurately identify those individuals with potentially significant pathology, these tests also need to be sensitive and specific to reduce overdiagnosis [10]. This may relate, in part, to the sample type being analysed. For example, the measurement of a biomarker in a faecal sample is more likely to be specific for early-stage colon cancer than the same biomarker measured in blood [11]. Another major consideration is the threshold at which a test is reported. 

CRC is essentially a heterogeneous disease comprising different molecular subtypes [12] that can be found in different anatomical locations [13] and at different stages [14]. Adding more complexity is the fact that while some biomarkers also detect precancerous lesions that include adenomas and sessile serrated polyps, the size and/or number of these lesions has the potential to impact on the test sensitivity, as does the level of dysplasia. Accordingly, a potential biomarker that detects early-stage disease in one individual may lack the sensitivity to detect disease in another. To address this, combinations of biomarkers are being investigated as a means to cover a range of host responses that may signal early-stage disease with the goal that this approach may more accurately identify those patients who should be referred for colonoscopy. 

The aim of this review is to consider a range of protein, metabolic, molecular and bacterial biomarkers (detailed in Table 1) for their ability to identify symptomatic patients presenting in primary care who should be progressed for clinical investigation. 

## 2. Faecal Haemoglobin

Of all symptoms, rectal bleeding is most strongly associated with CRC diagnosis, and testing for blood in faeces has been used to assess symptomatic patients in primary care for many years. Faecal immunochemical tests (FIT) for haemoglobin provide a higher sensitivity than the original guaiac test for faecal occult blood (FOB), and allow for quantification of faecal haemoglobin (f-Hb). However, a lack of standardisation makes comparisons across studies that report FIT difficult. Notably the reporting of f-Hb present in the collection or measurement solution (which can differ across different FIT products) rather than quantity of f-Hb present [15]. Less well appreciated with regards to the test itself is evidence of inter product variability [7,16], an issue that potentially gains even more significance as point-of-care testing increases [17]. The use of different f-Hb cut-off concentrations is another confounding factor. As f-Hb cut-off is increased, positivity rate, neoplasia detection rate and sensitivity decrease, while positive predictive value and specificity increase [18], as evidenced by two meta-analyses that indicate the widely varying sensitivity in a screening setting that reflects FIT threshold [7,19]. Collectively, these issues highlight the need for this test to be standardised, particularly in symptomatic patients where the first objective is to rule out CRC. When this is addressed, Westwood and colleagues [7] suggest that FIT has the potential to correctly rule out CRC and avoid colonoscopy in 75–80% of symptomatic patients. Based on these findings, the National Institute for Health and Care Excellence (NICE) diagnostic guidelines now recommend a threshold of 10 µg Hb/g faeces to guide referral for colorectal cancer in primary care [20].

There are a growing number of studies that report using FIT in symptomatic patients as a means to identify advanced colorectal neoplasia [21,22,23,24,25,26,27,28], and the diagnostic accuracy of FIT at a threshold of 10 µg Hb/g faeces in this setting is becoming clear (Table 2). A prediction model for CRC detection in symptomatic patients based on FIT concentration, age and sex may further aid diagnosis [29]. One concern, however, is the small proportion of patients found at colonoscopy to have CRC, despite no evidence of f-Hb, even when the cut-off for the test is reduced to the lowest limit of detection (f-Hb < 2 µg/g) [21,28]. This may, in part, reflect FIT measurement reportedly being significantly higher in left-sided colonic lesions compared with the right side [30,31]. One study that used a slightly higher FIT threshold (17 µg/g) found that approximately one--third of stage 1 CRCs were missed [31]. These studies highlight that while valuable as an adjunct to clinical history, FIT is not a diagnostic test in itself to identify all patients with early-stage disease [27].

## 3. Inflammatory Markers

Inflammation is considered a hallmark of cancer [32]. Neutrophils play a major role in the release of calprotectin at sites of inflammation, and faecal calprotectin (FC) has long been considered a potential biomarker of colorectal polyps and cancer [33]. However, FC is shown to have limited diagnostic accuracy for identifying patients with CRC, irrespective of stage [33,34,35,36,37] and this is reinforced by studies that have compared the sensitivity and specificity of FC to quantitative FIT in this setting [21,23,30,38,39]. 

The benefit of measuring FC levels in stool samples of patients who present in primary care with symptoms may instead be linked to the negative predictive value (NPV) of the test in a CRC diagnostic setting. Studies to date report NPV between 97.2–98.7 for CRC, and 93.2–97.2 for high-risk adenomas [21,37,39,40]. NICE guidelines accept a 3% risk in missing CRC in setting symptom criteria for referral [41], leading to the suggestion that FC concentrations below an established threshold may help rule out younger patients who more commonly present with nonspecific lower GI symptoms [42]. 

Another inflammatory marker gaining attention as a potential biomarker of early-stage CRC is chitinase-3-like protein 1 (CHI3L1), a glycoprotein produced by cells including macrophages, neutrophils and tumour cells. A large prospective study of patients referred for colonoscopy due to symptoms or other risk factors for CRC found serum CHI3L1 levels independently predict colon cancer in patients without comorbidity [43]. More recently, serum CHI3L1 was reported as having a high diagnostic value (96% sensitivity, 91.7% specificity) in diagnosing CRC, although no association with anatomical location or stage of the cancer was observed [44]. 

The mechanism(s) linking CHI3L1 to CRC are becoming clearer. This glycoprotein reportedly has multiple roles across normal cell growth, proliferation and survival within the colonic epithelium [45]. A finding of high levels of CHI3L1 in colonic biopsies from patients with IBD [46] and CRC [47] links CHI3L1 with inflammation, and this is confirmed by increased Interleukin (IL)-8 secretion in vitro when intestinal epithelial cells are engineered to overexpress CHI3L1 [47]. More recently, a positive correlation between tumour-associated CHI3L1 and IL-8 protein levels with tumour growth has also been reported [48]. Activation of major signalling pathways [49,50] that involve STAT3 signalling [51] are likely to underlie this response, orchestrated by the subsequent recruitment of macrophages to sites of inflammation [52]. Collectively, these preclinical and laboratory-based findings support further investigation of this inflammatory marker in a clinical setting. 

## 4. Intercellular Stability

E-cadherin is a calcium-dependent transmembrane glycoprotein key to the formation of adherens junctions between cells, and is considered to act as a signalling hub in colon homeostasis and disease [53]. Loss of E-cadherin function, which is associated with increased cell proliferation [54], can be linked to genetic mutations [55] and/or post-translational modification of the *CDH1* gene that codes for E-cadherin [56]. However, it may also signal a dysfunctional physiological response involving overexpression of cell-surface-associated proteases, leading to dysregulation of cell signalling pathways [57] and/or the presence of specific protease-producing bacterial species within the gut microbiota [58,59]. Irrespective of the mechanism involved, the resultant cleavage of extracellular E-cadherin results in the release of soluble (s)E-cadherin that is shown to bind to cell surface receptors and trigger downstream effects via different cell signalling pathways that have a role in the genesis of CRC [60,61]. 

Most studies appear to describe elevated sE-cadherin levels in patients with advanced and metastatic CRC, while levels are usually normal in early-stage disease. Velikova et al. [62] were the first to report elevated sE-cadherin levels in patients with local and metastatic disease, although no significant difference in sE-cadherin concentrations between patients with CRC and a group of healthy subjects was found. Likewise, Weiss et al. [63] reported significantly elevated levels of sE-cadherin in sera from patients with late Stage III and Stage IV carcinomas, and in patients with FAP, however the concentration of sE-cadherin in early-stage cancers did not differ significantly from healthy controls. 

In 2012, Okugawa et al. [64] showed increasing concentration of sE-cadherin across 186 colorectal cancers that were categorised by stage. Notably, the concentration of sE-cadherin increased significantly in patients presenting with Stage IV disease (9471 ± 7228) when compared to healthy controls (4509 ± 2709 ng/mL). More recently, Zhu et al. [65] confirmed and extended these findings. Significantly higher concentrations of sE-cadherin were detected in the patient cohort (*n* = 142) compared to serum levels in samples collected from 50 healthy controls (7184 ± 2931 ng/mL and 4317 ± 1687 ng/mL, respectively; *p* < 0.0001). Moreover, in this study, levels of sE-cadherin were found to correlate with UICC classifications I-II and III-IV (6654 ± 2556 ng/mL and 8130 ± 2767 ng/mL, respectively; *p* < 0.001). Further analysis determined the area under the receiver operating characteristic (ROC) curve of sE-cadherin was 0.853 for discriminating CRC from healthy controls, and that setting the optimal cut-off point for the ELISA at 5928 ng/mL resulted in diagnostic sensitivity and specificity of 73.9% and 80%, respectively. It is interesting to note that the mean sE-cadherin concentrations reported by Okugawa et al. [64] are very similar when the UICC classifications are similarly grouped (I-II and III-IV, 6431 ng/mL and 7971 ng/mL, respectively). In contrast however, an unrelated study that included 36 patients with CRC reported concentrations of sE-cadherin were as high in patients with benign tumours as in patients with CRC [66]. Measuring faecal sE-cadherin may improve the sensitivity of this test with regards to diagnosis of early-stage disease [11]. 

## 5. Metabolic Markers

Cancer is increasingly considered a metabolic disease as evidence emerges that many oncogenes and tumour suppressors play a key role in cellular metabolism. Three major metabolic pathways (aerobic glycolysis, glutaminolysis and one-carbon metabolism) are reportedly altered in CRC [67], and detection of functional changes in these pathways by the analysis of metabolites is increasingly considered as a means to diagnose early-stage disease [68]. 

The dimeric M2 isoform of pyruvate kinase (also known as tumour M2-pyruvate kinase and M2-PK), an organ-unspecific biomarker that reflects the metabolic activity and proliferation capacity of any tumour per se [69,70], is one example. Moreover, since M2-PK is the direct target of several oncoproteins including K-ras [71], detection of faceal M2-PK has been suggested to be a more sensitive screening tool for CRC than detection of mutations in the oncogenes themselves [72]. Whereas this approach showed early promise with regards to M2-PK levels as a biomarker of CRC [72], and also potentially disease staging [38,73], variable levels of specificity in subsequent studies have questioned the utility of this biomarker [38,74,75].

This is highlighted by three systematic reviews and meta-analyses of the diagnostic accuracy of faecal M2-PK that found varying levels of sensitivity and specificity (79.0%–80.3% and 80.0%–95.2%, respectively) [76,77,78]. These findings are reinforced by faecal M2-PK failing to identify precancerous bowel lesions or CRC in a subset of symptomatic patients [79]. M2-PK also has reduced diagnostic sensitivity and specificity for detecting early-stage disease when compared to f-Hb [74,75,78,79]. It is possible however that this relates, in part, to the assay used to determine M2-PK levels. Tumour cells readily switch between the dimeric and less active tetrameric form of M2-PK to give themselves metabolic flexibility in response to environmental changes [69,70,80,81], whereas the assay only measures the dimeric form of the enzyme [74]. 

Finding metabolic markers that can risk stratify patients with suspected CRC is an area of active investigation. These markers broadly divide into two groups, volatile organic compounds (VOCs) and small metabolites that can be detected using urine, blood and faecal samples. VOCs are also detected in exhaled breath [14,82]. The potential diagnostic accuracy of VOCs for early detection of CRC, exemplified by the sensitivity and specificity of canine scent detection [83], is now reflected in studies that report VOCs in the headspace of urine [23,84,85], breath [86,87,88,89], blood [90] and faeces [91,92,93,94] (Table 3). There is also evidence that detection of urinary VOCs can improve CRC detection in FIT-negative patients [23]. The development of electronic nose technology [84] may bridge this approach with clinical practice in the future.

The growing number of studies looking for nonvolatile CRC-associated metabolites suggests these will also be used in the future to triage symptomatic patients [14,95,96]. However, this approach will not be without challenges. Interindividual differences that reflect host-specific diets and/or gut microbiota will need to be considered [2,97,98], and already a quantitative approach and/or the use of metabolite panels is being reported [99,100]. Secondly, as identified elsewhere, the cost and complexity of these assays will need to be addressed before this approach is introduced into routine clinical practice [14].

## 6. Genetic Markers

Most cases of CRC are due to sporadic genetic and/or epigenetic changes, and it is these changes that drive carcinogenesis down pathways resulting in different disease phenotypes that include microsatellite instability (MSI), chromosomal instability (CSI), and the CpG island methylator phenotype (CIMP). This means that the substantial genetic heterogeneity inherent in CRC needs to be considered when developing molecular diagnostic methods. 

Whereas identifying gene mutations alone in faecal DNA lacks diagnostic accuracy [101], testing for gene-specific methylation is more promising, which may relate to epigenetic changes that occur at an early stage in the precancerous pathway [102]. This has led to development of two commercial assays. The first detects aberrantly methylated *BMP3* and *NDRG4*, mutant *KRAS*, β-actin and haemoglobin in a single faecal sample and has a sensitivity of 92.3% and 69.2% for detecting CRC and advanced precancerous lesions, respectively [103]. The test, which is marketed as Cologuard^®^ in the USA, is technically complex and costly. However, it is more sensitive and specific than FIT alone [103]. The second commercial assay, which is designed to detect methylated *SEPT9* in blood, may also be useful in a diagnostic setting [104], but additional studies are needed to confirm this.

One major limitation of this approach however is that it would need to be broad enough to detect early-stage disease (including premalignant (dysplastic) lesions) in all individuals. Virtually all CRCs have a subset of multiple alterations that drive the initiation and progression of precancerous changes through processes that include the accumulation of epigenetic changes [105]. These changes may be accelerated via the production of bacterial metabolites [106], thus reflecting an individual’s gut microbiome and/or diet. The rate of DNA methylation also changes with age, and is reportedly side- and race-specific [107]. Accordingly, epigenetic markers may have greater utility for indicating potential pathways to CRC subtype formation [12,105,108] as opposed to being considered as a means to identify patients presenting in primary care who should be progressed for clinical investigation. 

Micro (mi)RNAs are a subclass of small, non-coding RNAs that are gaining attention as molecular biomarkers of early-stage CRC [109]. However, while many have been investigated in this setting [14], some show more promise than others [110]. The concept that a panel of miRNAs will collectively have greater diagnostic accuracy is one area of active investigation [111]. 

## 7. Gut Microbiota

The gut microbiota is increasingly considered to have a role in influencing the biology of CRC, and this association is demonstrated using a number of different approaches. The simplest is screening faecal samples for molecular evidence of known bacterial virulence factors considered to have a role in initiating CRC. Enterotoxigenic *Bacteroides fragilis* (ETBF) express a toxin [112] that is associated with promotion of carcinogenesis in mice [113] and humans [114], However, faecal ETBF lacks the specificity and sensitivity needed of a reliable biomarker in a diagnostic setting [115,116]. 

Another approach considers the environmental changes in early-stage disease that allow some bacterial species to outcompete others [117], resulting in the development of dysbiosis. This reflects shifts in the relative abundance of some members of the gut microbiota and is associated with progression to adenoma [118,119], adenoma to carcinoma [120,121] and CRC [122,123]. Screening for specific taxonomic bacterial markers reveals *Fusobacterium nucleatum* is significantly increased in faecal samples from patients presenting with adenomas and CRC when compared to healthy controls [124,125,126,127]. Moreover, these bacteria are functionally linked to colorectal carcinogenesis [124,128,129,130]. While it may be possible to predict colon tumorigenesis on the basis of a CRC-associated signature such as molecular evidence of faecal *F. nucleatum*, this approach does not account for microbial community dynamics [131] that are informed by factors including diet, repeated antibiotic exposure, stress and/or exposure to pathogens [132,133,134]. Additionally, host miRNAs may also affect the growth and composition of the gut microbiota [135]. Long-term, these environment-and/or host-associated changes can result in a shift in bacterial abundance where some species are better or less able to use limiting resources than others [136], as evidenced by the analysis of bacterial metabolites [96,98] that, in turn, may contribute downstream to epigenetic modification [106]. 

## 8. Combinations of Biomarkers

Assaying for the presence of fHb in stool samples is widely considered the gold standard test to identify symptomatic patients who should be progressed to clinical investigation. However, even at a low threshold, the FIT test still does not identify all symptomatic patients presenting with early-stage disease. Combinations of biomarkers are increasingly seen as a way to address this. For example, the Cologuard test that screens for molecular markers of early stage disease also includes the measurement of faecal haemoglobin [103]. A comparison of the Cologuard test versus FIT alone clearly shows the increased diagnostic accuracy with this combined approach (Table 4).

There are other studies that also show diagnostic accuracy is increased when a second biomarker is used in addition to FIT (Table 4). For example, measuring M2-PK in faecal samples associates this biomarker with CRC (sensitivity, 87%, NPV, 96%) at a cut off of 4 U/mL. However, when tested over a range of concentrations (4, 10 and 15 U/mL), any positive test combined with FIT resulted in the overall sensitivity increasing to 91.5%, with an NPV of 97% [38]. The recent study by Cruz et al. [137] confirms this approach has greater diagnostic accuracy than reporting either FIT or M2-PK alone. Screening for urinary VOCs is also shown to improve detection of CRC in symptomatic patients who are FIT-negative [23] (Table 4).

Individual bacterial species may likewise signal risk, as illustrated by Baxter et al. [138] who showed detection of colonic lesions increased when the relative abundance of certain strains of gut microbiota was assessed in combination with FIT. This study suggested an association of *F. nucelatum* with CRC, and subsequent studies confirm quantitation of *F. nucleatum* in faecal samples in combination with FIT increases diagnostic accuracy over FIT alone in detecting CRC [126] and advanced adenomas [127] (Table 4). In contrast, detecting FC in combination with FIT appears to add little or no additional diagnostic information [21,23,38,39] (Table 4).

## 9. Concluding Remarks

An FIT with a threshold of 10 ug Hb/g faeces clearly has utility as a first-line test for identifying symptomatic patients who should be progressed for definitive clinical investigation. However, this test does not detect everyone with early-stage disease, and, as detailed above, there are a growing number of molecular, protein and chemical based approaches that could be tested in combination with FIT to increase diagnostic accuracy. What is becoming apparent is the interconnectedness between many of these biomarkers, as evidenced by CHI3L1-mediated suppression of E-cadherin expression [139], microbiota-derived metabolite involvement in epigenetic alteration [140], and the effect of miRNA in the growth and composition of the gut microbiota [141], which may contribute to the reported role of miRNA-associated colon carcinogenesis [110]. This is a rapidly evolving area of research, and in the future it is likely that combinations of biomarkers may be able to detect dysplastic lesions that can be treated at a low cost to both the patient and health providers.

**Table 1:** Noninvasive biomarkers.

Indicator Type	Biomarker	References
Tissue damage	Faecal haemoglobin	[7,33,34,35,36,37,38,39,40,41,42,43,44,45,46,47,48,49,50,51]
Inflammation	Calprotectin	[21,23,30,33,34,35,36,37,38,39,40,41,42]
	Chitinase 3-like 1	[43,44,45,46,47,48,49,50,51]
Intercellular stability	E-cadherin	[53,54,55,56,57,58,59,60,61,62,63,64,65,66]
Metabolic	M2-pyruvate kinase	[38,69,70,71,72,73,74,75,76,77,78,79,80,81]
	Volatile organic compounds	[14,23,82,83,84,85,86,87,88,89,90,91,92,93,94]
	Small metabolites	[14,95,96,97,98,99,100]
Host genetics	Genetic and/or epigenetic markers	[102,103,104]
	MicroRNA	[14,105,106,107]
Gut microbiota	*Fusobacterium nucleatum*	[120,121,122,123,124,125,126]

**Table 2:** Diagnostic characteristic of FIT ≥ 10 µg Hb/g faeces in symptomatic patients.

Study	*n*	Disease	Sensitivity (%)	Specificity (%)	PPV	NPV
Mowat et.al. 2016 [21]	1043	CRC (*n* = 28)	89.3	79.1	14.2	99.5
		HRA (*n* = 41)	50.0	78.0	11.4	96.5
Widlak et al. 2018 [23]	562	CRC (*n* = 35)	80	93	44	99
		HRA (*n* = 27)	63	76	11	98
D’Souza et al. 2021 [28]	9822	CRC (*n* = 329)	90.9	83.5	16.1	99.6
		HRA (*n* = 421)	45.4	82.2	10.3	97.1

FIT, faecal immunochemical test for haemoglobin; PPV, positive predictive value; NPV, negative predictive value; HRA, high-risk adenoma.

**Table 3:** Volatile organic compounds (VOCs) used for CRC and advanced adenoma detection.

Study Setting	Sample Type	Biomarker(s)	Sensitivity (%)	Specificity (%)	Ref.
Case–control	Stool	VOC patterns—detected by electronic nose	85 (CRC) 62 (AA)	87 (CRC) 86 (AA)	[91]
FOBT+	Stool	Hydrogen sulphide, dimethyl sulphide, dimethyl disulphide—detected by SIFT-MS	72	78	[92]
Case–control	Stool	Methyl mercaptan, hydrogen—detected by GC	90	57.7	[94]
Case–control	Stool	Propan-2-ol, hexan-2-one–detected by GC-MS	87.9	84.6	[93]
Case–control	Urine	VOC fingerprint—detected by FAIMS	88	60	[85]
Case–control	Urine	VOC fingerprint—detected by FAIMS	63	63	[23]
Case–control	Breath	Acetone, ethylacetate, ethanol, 4-methyl-octane—detected by GC-MS	85	94	[86]
Case–control	Breath	Nonanal, decanal, 4-methyl-2-pentanone, 2-methylbutane, 4-methyloctane, 4-methylundecane, 2-methylpentane, 3-methylpentane, methylcyclopentane, cyclohexane, methylcyclohexane, trimethylcyclohexane-1,2-pentadiene, 1,3-dimethylbenzne, 1,4-dimethylbenzene–detected by GC-MS	86	83	[88]
Case–control	Breath	cyclohexanone, 2,2-dimethyldecane, dodecane, 4-ethyl-1-octyn-3-ol, ethylaniline, cyclooctylmethanol, trans-2-dodecen-1-ol, 3-hydroxy-2,4,4 trimethylpentyl 2-methylpropanoate, 6-t-butyl-2,2,9,9-tetramethyl-3,5-decadien-7-yne—detected by SPME-GC/MS			[87]
Colonoscopy patients	Breath	VOC patterns—detected by electronic nose	95% (CRC) 79% (AA)	64% (CRC) 59% (AA)	[89]

CRC, colorectal cancer; AA, advanced adenoma, defined as ≥10 mm, with any villous features or high-grade dyspasis); FOBT, faecal occult blood test. SIFT, selected ion flow tube; MS, mass spectrometry; GC, gas chromatography; FAIMS, field asymmetric ion mobility spectrometry; SPME, solid-phase microextraction.

**Table 4:** Diagnostic performance of biomarker combination for CRC and high-risk/advanced adenoma.

Disease Group	Biomarker(s)	Sensitivity (%)	Specificity (%)	PPV (%)	NPV (%)	Ref.
CRC (*n* = 65)	FIT ^a^	73.8	94.9			[103]
	Cologuard^®^	92.3				
HRA (*n* = 757)	FIT ^a^	42.2	86.6			
	Cologuard^®^	23.8				
CRC (*n* = 18)	FIT ^a^	61.7	88.8	52.7	92	[38]
	FIT and M2-PK ^d^	91.5	57.1	30.1	97.1	
AA (*n* = 28)	FIT ^a^	35.6	94.5	85.4	62.2	
	FIT and M2-PK	71.2	66.9	65.7	72.3	
CRC (*n* = 60)	FIT ^b^	91.5	72.3			[134]
	FIT and M2-PK ^e^	49.1	93.5			
	FIT or M2-PK	96.6	58.2			
CRC (*n* = 35)	FIT ^c^	80	93	44	99	[23]
	VOCs (FIT –ve)	97	72		100	
CRC (*n* = 104)	FIT ^a^	73.1	98			[123]
	FIT and *Fn.*	92.3	93			
AA (*n* = 103)	FIT ^a^	15.5	98			
	FIT and *Fn*	38.6	89			
CRC (*n* = 18)	FIT ^a^	61.7	88.8	52.7	92	[38]
	FIT and/or FC ^f^	95.7	26.4	22.1	96.6	
AA (*n* = 28)	FIT ^a^	35.6	94.5	85.4	62.2	
	FIT and/or FC	82.8	26.9	49.5	64.4	
CRC (*n* = 35)	FIT ^c^	80	93	44	99	[23]
	FIT and FC	80	93	43	99	
HRA (*n* = 27)	FIT ^c^	63	76	11	98	
	FIT and FC	93	25	6	99	
CRC (*n* = 28)	FIT ^a^	89.3	79.1	14.2	99.5	[21]
	FIT and/or FC ^g^	100	20.3	4.7	100	
HRA (*n* = 41)	FIT ^a^	50	78	11.4	96.5	
	FIT and/or FC	92.7	20.3	6.3	97.7	
CRC (*n* = 16)	FIT ^a^	87.5	83.7	18.2	99.4	[39]
	FIT and FC^g^	93.8	43.3	6.4	99.4	
AA (*n* = 39)	FIT ^a^	46.2	83.8	23.4	93.6	
	FIT and FC	82	44.4	13.6	98.9	

HRA, high-risk adenomas; AA, advanced adenomas; Faecal immunochemical test (FIT) cut-off: ^a^, 20 ug; ^b^, 10 µg; ^c^, 3 µg f-Hb/g stool; M2-PK cut-off values: ^d^, 4, 10, & 15 U/mL; ^e^, 8 U/mL; Faecal calprotectin (FC) cut-offs: ^f^, 50 and 416 µg/g; ^g^, 50 µg/g; VOCs, volatile organic compounds; Fn, *Fusobacterium nucleatum.*

## Data Availability

Not applicable.
